# Durability of Structural Lightweight Concrete with Sintered Fly Ash Aggregate

**DOI:** 10.3390/ma13204565

**Published:** 2020-10-14

**Authors:** Lucyna Domagała

**Affiliations:** Faculty of Civil Engineering, Cracow University of Technology, 31-155 Cracow, Poland; ldomagala@pk.edu.pl

**Keywords:** durability, lightweight concrete, lightweight aggregate, sintered fly ash, moisture content, compressive strength, water absorption, water permeability, freeze-thaw resistance, microstructure

## Abstract

The aim of this study was to present the problem of durability of structural lightweight concrete made of a sintered fly ash aggregate. The issue of durability was researched for 12 concrete series in terms of their water absorption, water permeability, and freeze-thaw resistance. Additionally, the microstructure of several concretes was analyzed with a scanning electron microscope (SEM). In the durability research, the influences of the following parameters were taken into consideration: The initial moisture content of sintered fly ash (mc = 0, 17–18, and 24–25%); the aggregate grading (4/8 and 6/12 mm); and the water-cement ratio (w/c = 0.55 and 0.37). As a result of various compositions, the concretes revealed different properties. The density ranged from 1470 to 1920 kg/m^3^, and the corresponding strength ranged from 25.0 to 83.5 MPa. The durability research results of tested lightweight concretes showed that, despite considerably higher water absorption, a comparable water permeability and comparable or better freeze-thaw resistance in relation to normal-weight concrete may be present. Nevertheless, the fundamental requirement of lightweight concrete to achieve good durability requires the aggregate’s initial moisture content to be limited and a sufficiently tight cement matrix to be selected. The volume share of the cement matrix and aggregate, the cement content, and even the concrete strength are of secondary importance.

## 1. Introduction

Contemporary structural lightweight aggregate concrete (LWAC) is a building material that is widely used in civil engineering. In many cases, LWAC may be treated as a convenient alternative to structural normal-weight aggregate concrete (NWAC). It is especially used in structures where longer span elements, a lower dead load, and better thermal insulation are required. Therefore, lightweight aggregate concrete is applied for both precast and monolithic constructions, in particular, high rise and public buildings, sports and entertainment halls, stadiums, car parks, bridges and viaducts, roads, tunnels, tanks, oil rigs, and many other engineering structures.

It is not without significance that LWAC may be considered as a structural building material that follows the principles of sustainable development much better than NWAC. Firstly, many types of structural lightweight concrete are prepared with manufactured aggregates made of by-products, e.g., sintered fly ash, blast furnace slag, or recycled glass. Secondly, the better thermal insulation of LWAC promotes a lower energy consumption, resulting from heating and air conditioning during building operations. Thirdly, the possible better durability of structural lightweight concrete in comparison to NWAC significantly contributes to sustainability due to the lower costs of construction, maintenance, and repair.

### 1.1. Specificity of Lightweight Aggregate Concrete’s Durability

Generally, the possible better durability of LWAC may result from its better structural homogeneity. It is connected to a manufactured lightweight aggregate of a more regular size and shape, internal curing with water accommodated in the lightweight aggregate (LWA), the better material compatibility of the main composite components (porous cement matrix and porous aggregate), and their better bonding [1,2,3]. The bonds between the lightweight aggregate and cement paste may be built by mechanical inter-locking; the absorption of water/cement paste from fresh concrete by the aggregate; or, in some cases, the pozzolanic reactivity of several LWAs [4,5,6,7,8,9,10]. As a result of the better structural homogeneity, structural lightweight concrete usually reveals less cracking due to shrinkage, creep, thermal deformation, or loads [6,11,12,13]. Therefore, in many cases, LWAC may perform in construction in an uncracked condition. Meanwhile, this essential fact, related to the durability of LWAC, is not considered in standard durability tests, since they are carried out on relatively small, unloaded specimens. As a result, the tests do not show the full durability potential of structural lightweight concrete.

Nevertheless, it should be noted that the durability of structural lightweight concrete is a very complicated problem and the influence of material and technological factors on LWAC’s performance may be stronger and more complex than in the case of NWAC. That is why there are so many conflicting reports on the subject of lightweight concrete’s durability. As a rule, there is no doubt that LWAC has a higher fire resistance in relation to normal-weight concrete [2,3,14,15,16]. Research on water permeability, chloride penetration resistance, freeze-thaw resistance, and carbonation does not give such an unequivocal trend. In this case, the main reasons for the divergence in research results are probably differences in aggregate properties, in particular, in the porosity structure and water absorption of LWA, as well as the various procedures of concrete preparation used, including initial aggregate pre-wetting.

### 1.2. Water Tightness of Lightweight Aggregate Concretes

Despite the usually higher water absorption of lightweight concretes, their water tightness may be comparable to, and even better than, normal-weight concretes. This has been confirmed by numerous studies, e.g., [17,18,19,20], which showed a similar depth of water penetration under pressure for LWAC and NWAC when a relatively tight cement matrix (water to binder ratio w/b ≤ 0.4) and aggregates with relatively low water absorption (WA_24h_ ≤ 10–15%) were used. However, as demonstrated by Liu et al. [19], the use of a very tight cement matrix (w/b = 0.20) made even lightweight concrete prepared with aggregates of high water absorption (expanded clay of WA_24h_ = 12–30%; expanded glass of WA_24h_ = 28–52%) practically waterproof. In tests of concrete with a pumice aggregate, Hossain et al. [20] revealed that replacing a normal-weight aggregate with a lightweight aggregate with water absorption higher than that of most artificial aggregates (WA_24h_ = 27–32%), in the range of 0 to 100%, resulted in a decrease in the water permeability of concrete. Nevertheless, the increase in the LWA content in relation to cement paste, regardless of its type, caused an increase in water permeability. A similar effect was achieved by the replacement of natural sand with its light counterpart, which was demonstrated by Liu et al. [19]. According to Zhang and Gjorv [21], the type and amount of cement and mineral additives also affect the water permeability of lightweight concrete. In this regard, the optimal cement content in lightweight concrete turned out to be approximately 500 to 600 kg/m^3^. Outside the indicated range of the cement content, a decrease in the water tightness of lightweight concretes was observed. Moreover, a higher water depth of penetration of LWAC, in relation to NWAC with a comparable composition, is more likely when open pore structure aggregates, e.g., cold bonded LWAs, are used [22].

### 1.3. Chloride Penetration of Lightweight Aggregate Concretes

In the case of chloride penetration resistance, there have been many studies on LWAC showing its lower or comparable penetration of chloride ions in comparison to NWAC of the same strength, even for ordinary concretes with a similar composition (e.g., [10,17,19,20,21,23]). However, there are also some research examples that have indicated a higher concentration of chloride ions in lightweight concretes (e.g., [24,25]). Nevertheless, even in these cases, an increased corrosion of reinforcing steel was not confirmed, which should probably be attributed to the good adhesion of lightweight concrete to the reinforcement. In the general case of chloride penetration tests of LWAC with good bonding between LWA and cement paste, as occurs in water permeability tests, the type of lightweight aggregate is less important than the tightness of the cement matrix itself. As Nadesan and Dinakar showed in their review [10] dedicated to sintered fly ash concretes, the less pronounced permeability and chloride penetration of LWACs in comparison to normal-weight aggregate concretes should be attributed to their superior interfacial transition zone. Bogas and Real, in their review [25] of both chloride penetration resistance and carbonation, stated that the most important factors influencing the different tendencies reported in the literature are the paste composition, type of aggregate, curing and exposure conditions, test setup, penetration mechanism, and concrete water content. Zhang and Gjorv [21] additionally showed that differences in the assessment of the chloride ion permeability in lightweight concretes resulted not only from the use of various research procedures, but also from the specifics of the tests themselves, which caused significant dispersion of the results. 

### 1.4. Carbonation of Lightweight Aggregate Concretes

Due to its higher gas permeability, related to its much higher porosity, LWAC may reveal a greater depth of carbonation in comparison to NWAC. Additionally, the susceptibility of lightweight concrete to carbonation may be increased by a higher moisture content owing to the huge amount of water accommodated in the porous aggregate. Therefore, many researchers have reported more pronounced carbonation for lightweight concrete [25]. Despite these facts, there is also significant evidence that LWAC, especially when it consists of a relatively tight cement matrix and aggregates displaying relatively low water absorption, is able to obtain a comparable or even smaller depth of carbonation in relation to NWAC of the same strength class [2,23,24,26].

### 1.5. Freeze-Thaw Resistance of Lightweight Aggregate Concretes

The unambiguous assessment of freeze-thaw resistance of structural lightweight concrete in comparison to NWAC is even more difficult than in the case of the other properties related to durability mentioned above. On the one hand, many published works (e.g., [10,18,27,28,29]) have shown that lightweight concretes, even when air entraining admixtures are not used, may be characterized by a higher frost resistance compared with normal-weight concretes of the same strength classes. This has also been confirmed by assessments of the state of concrete in existing structures exposed to cyclic freezing and thawing [30,31]. Moreover, it was proven in [27,32] that, by using dry aggregate, it is possible to achieve a comparable or much better frost resistance of lightweight concretes compared to ordinary concretes, even in the case of concretes having the same volume composition. By replacing the normal-weight aggregate, in part or even in entirety, with several types of LWA (Leca 670, Leca 800, Liapor 8), it is possible to improve the frost resistance. In the case of weaker and more porous pumice and Lytag aggregates, the increase in their content in relation to the share of the ordinary aggregate caused a greater decrease in strength after freeze-thaw resistance tests. On the other hand, Lotfy at al. [14], as well as Tang and Brouwers [22], revealed that, despite the porous nature of the LWAs used (expanded clay, expanded shale, furnace slag, and cold bonded LWAs) and relatively low water to binder ratio (0.28–0.40), non-air entrained LWACs exhibited a low resistance to freeze-thaw cycles. It should be noted that in the case of tests reported in [14,22], the aggregates were pre-soaked for at least 24 and 72 h, respectively. Fujiki et al. [28], as a condition for obtaining a higher freeze-thaw resistance of LWAC in relation to NWAC, indicated the degree of aggregate pre-wetting as being at a level of less than 90% of the total porosity of the concrete; otherwise, if the external conditions of concrete curing prevent its proper drying, the structure of concrete may be destroyed during freezing. Research by Klieger and Hansen [18] showed that the durability coefficient for some lightweight concretes with an initially dry aggregate can even be several times higher compared with concretes with a saturated aggregate.

In sum, the analysis of the reported research results indicates that LWAC may perform in a similar way to, or better than, NWAC when it is subject to water exposure under pressure, chloride attack, or freeze-thaw cycles, but under the condition that the used aggregate is characterized by a relatively low level of water absorption and the total water content in fresh concrete, resulting from both the cement paste composition and aggregate initial moisture content, it is considerably limited. Meanwhile, in practice, lightweight aggregates, especially those with high water absorption, are usually used for LWAC in a pre-saturated condition, in order to prevent workability loss and aggregate segregation in fresh concrete.

The aim of the research was to recognize the influences of the composition and preparation technology used for structural lightweight concrete made of a sintered fly ash aggregate with a relatively high water absorption rate on LWAC’s durability. In relation to the reported tests dedicated to concrete with this type of aggregate, the aim of this research was to investigate LWAC’s durability more comprehensively through many aspects at the same time, including the concrete density, strength, water absorption, water permeability, and freeze-thaw resistance, as well as several material and technological factors. In particular, the problem of sintered fly ash aggregate pre-saturation application was considered.

## 2. Materials and Methods

Twelve concrete mixtures were prepared for the tests. They differed in terms of their nominal water–cement ratio (w/c = 0.55 or 0.37), coarse aggregate grading (4/8 or 6/12 mm), and initial moisture condition of coarse aggregate (oven-dried (D), moistened (M), or water saturated (S)). Due to the ability of the porous aggregate, which was not initially saturated, to absorb water from cement paste, the effective w/c ratios of fresh lightweight concrete were certainly lower than the assumed nominal values. Nevertheless, as proved in [9], the effective water–cement ratio is very difficult to determine reliably and certainly should not be calculated only on the basis of the aggregate water absorption.

### 2.1. Constituent Materials

**Figure 1:**
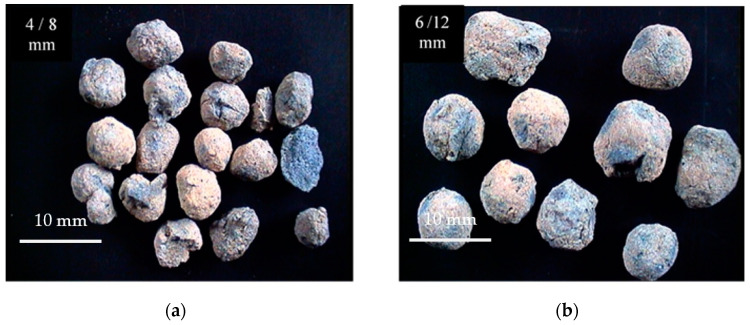
Sintered fly ash aggregate: (**a**) fraction 4/8 mm and (**b**) fraction 6/12 mm.

**Figure 2:**
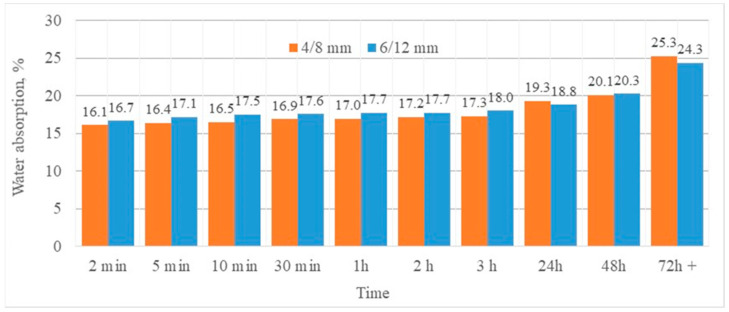
The development of water absorption of a sintered fly ash aggregate over time.

As a coarse aggregate, Lytag, lightweight sintered fly ash manufactured in Poland, was used (Figure 1). The aggregate was manufactured by sintering fly ash with the addition of fine coal at the temperature of approximately 1250 °C on a sintering grate. The basic properties of the sintered fly ash aggregate used, such as the specific, particle, and bulk density; water absorption; and bulk crushing resistance, specified according to the European Standards EN 13055-1 [33], EN 1097-3 [34], and EN 1097-6 [35], are presented in Table 1. The chemical composition of both the applied aggregate and cement is given in Table 2. Due to the relatively high bulk crushing resistance, the aggregate was deemed to be one of the most suitable for structural lightweight concretes. The aggregate water absorption, after immersion in water for 72 h, reached 24.3% and 25.3%, respectively, for the 6/12 and 4/8 mm fractions, and no longer increased. These values were used as the moisture contents of fractions for concrete in water-saturated conditions. However, a moistened condition of the aggregate meant that its moisture content corresponded to LWA water absorption after immersion in water for 1 h, which was 17.0% for the 4/8 mm fraction and 17.7% for the 6/12 mm fraction. The development of water absorption over time for selected fractions of the sintered fly ash aggregate is presented in Figure 2.

The rest of the constituent materials of concrete mixtures were Portland cement CEM I 42.5 R (see Table 2), natural sand as a fine aggregate, and tap water. Additionally, for mixtures with a lower water–cement ratio, superplasticizer (Sika ViscoCrete 3) was dosed. 

### 2.2. Concrete Compositions and the Preparation Procedure

The concretes were prepared in two stages: Firstly, a cement matrix in the form of mortar of a specified nominal water–cement ratio was made, and was then added to a lightweight aggregate of a specified moisture content condition in a certain amount, to allow a workable consistency of V3 to be reached; this was determined according to EN 12350-3 [36]. Therefore, the proportions of coarse aggregate and cement matrix were changeable for different concrete series and were mainly determined by the initial moisture content, which affected the capacity of the aggregate to absorb water from fresh concrete. The nominal water–cement ratio determined this proportion to a lesser degree. As a result, mixtures prepared with aggregates of a lower moisture content and/or with a lower water–cement ratio were characterized by a greater share of cement matrix in their compositions. The ratio of natural sand to cement was constant for all mixtures and was 1.20. The parameters and compositions of prepared concretes are given in Table 3 and Table 4.

### 2.3. Molding Specimens

All concretes were molded and compacted on a vibration table in the following forms: Cubes with sides of 100 mm and cubes with sides of 150 mm. After 24 h, all specimens were demolded and then stored in a climatic chamber (RH = 100%, T = 20 °C) for 27 days, according to EN 12390-2 [37]. Then, the specimens were prepared for the following tests: Oven-dried density; density under saturated conditions; water absorption; compressive strength under oven-dried and saturated conditions; the depth of water penetration under pressure; and freeze-thaw resistance. The tests conducted, the types of concrete specimens and their number, the concrete age for the tests, and the standard test procedures are listed in Table 5. Additionally, for comparison reasons, for some tests, reference specimens of plain mortars of w/c = 0.55 (matrix 1) and 0.37 (matrix 2), which were used as cement matrixes for both concrete series 1 and 2, were employed.

### 2.4. Procedures for Testing Hardened Concrete

The testing procedures used for the determination of the density, compressive strength, and water permeability are not described here, as they were conducted in accordance with the well-known European Standards EN 12390-7 [38], EN 12390-3 [39], and EN 12390-8 [40]. Since there are no European Standards dedicated to the determination of the water absorption of concrete or its freeze-thaw resistance, these tests were carried out according to Polish Standards PN-88/B-06250 [41] and PN-B/06265 [42]. 

The test of the water absorption of concrete consists of the saturation of three concrete specimens in water until their weights stabilize, determination of the mass of saturated specimens, drying the specimens under oven-dried conditions at a temperature of 105 °C, and determination of the mass of oven-dried specimens. Water absorption was determined as the water content in a saturated specimen relative to the oven-dried specimen’s weight, expressed as a percentage. 

**Figure 3:**
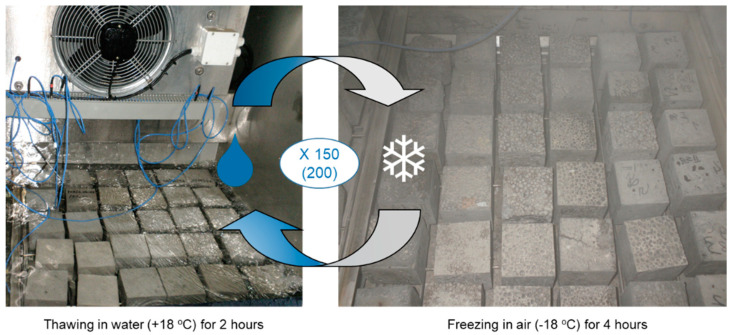
The freezing and thawing chamber with lightweight aggregate concrete (LWAC) specimens.

The test of freeze-thaw resistance began with the saturation of twelve concrete specimens in water until their weights stabilized. Six specimens were used as references and the other six were subject to freeze-thaw cycles. Freezing was carried out in air at −18 ± 2 °C for at least 4 h, while thawing took place in water at +18 ± 2 °C for 2–4 h. For this research, the number of cycles was assumed to be as high as 150, which is the typical value for concrete structures and structural members subject to freeze-thaw attack, including conditions of water capillary action, the fluctuation level of water, and the possibility of de-icing agent application. In the case of one concrete series (2d), which did not reveal any signs of frost damage after 150 cycles, an additional 50 cycles of freezing and thawing were carried out on half of the specimens. The scheme of freeze-thaw cycles used in this research is presented in Figure 3. After the freezing and thawing cycles, the appearance of the specimens was assessed for cracks, and the weight and strength losses were determined. Concrete was regarded as fulfilling the criteria for freezing and thawing resistance at an assumed number of cycles when it did not reveal any cracks, the weight loss was lower or equal to 5%, and the strength loss was not greater than 20%.

Additionally, the microstructure of selected LWAC (1D, 1M, 1S, 2D, 2M, and 2S) was analyzed under a scanning electron microscope (SEM), in order to recognize the fracture mechanism for lightweight concretes made with aggregates with different initial moisture contents.

## 3. Results

The basic properties of the tested concretes, i.e., the density and compressive strength in oven-dried and water saturated conditions, are presented in Table 6. As predicted, as the result of various compositions, the concretes revealed different properties. Their densities ranged from 1470 to 1920 kg/m^3^ and their corresponding strengths ranged from 25.0 to 83.5 MPa. The test results for density differed by no more than 20 kg/m^3^ from the average value specified for a given series. The coefficient of variation of compressive strength, defined as the ratio of the standard deviation and the mean strength value, was 0.05 on average for both cement matrices, as well as for all lightweight concretes, irrespective of whether the determination was carried out on specimens in dry or saturated conditions. For concretes prepared with an initially saturated aggregate (1s, 1S, 2s, and 2S), the coefficient was slightly higher (0.06–0.07) than for the rest of the composites (0.04–0.06). Nevertheless, such low coefficient of variation values testify to the very good strength homogeneity of all tested concretes and their matrices.

According to EN 206 [43], all tested concretes may be classified as structural and lightweight. Their strength classes ranged from LC16/18 up to LC60/66 and their density classes ranged from D1.6 to D2.0.

### 3.1. Water Absorption

The mean results of the water absorption tests are given in Table 6. The values ranged from 5.6% up to 21.9%. The dispersion of results was very low, and individual results mostly differed from the average values of water absorption by no more than 0.1 percentage point. Despite the application of a lightweight aggregate characterized by considerable water absorption, the results of concrete water absorption (WA) may be regarded as at least satisfactory. According to the requirements given in [40], all tested concretes met the criterion for lightweight concrete performed under conditions of protection from contact with atmospheric factors (WA < 25%). In the case of unprotected concrete exposed to direct weather conditions, the more rigorous criterion of less than 20% water absorption was only not met by the 1s concrete.

### 3.2. Water Permeability

**Figure 4:**
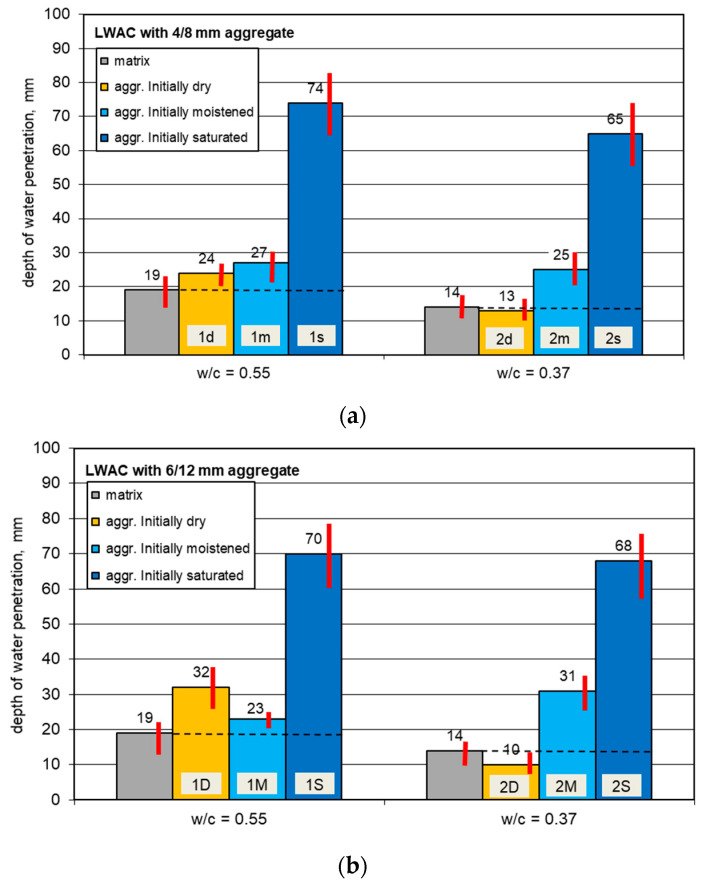
The impact of the aggregate’s initial moisture condition, aggregate size, and cement matrix on the depth of penetration of water under pressure in LWAC: (**a**) LWAC with aggregate 4/8 mm and (**b**) LWAC with aggregate 6/12 mm.

**Figure 5:**
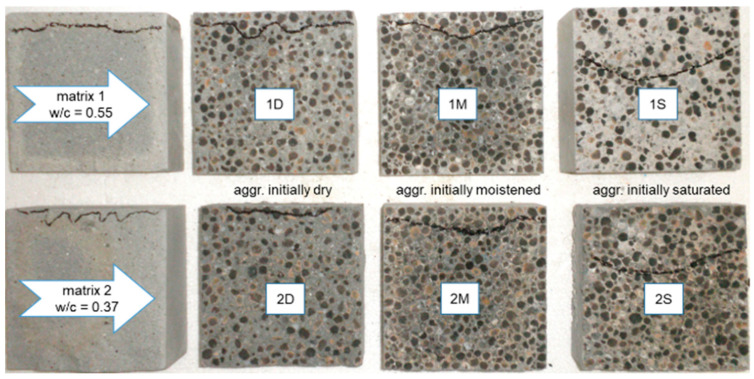
The appearance of specimens of mortars and concretes split after water permeability tests, with marked water fronts.

**Figure 6:**
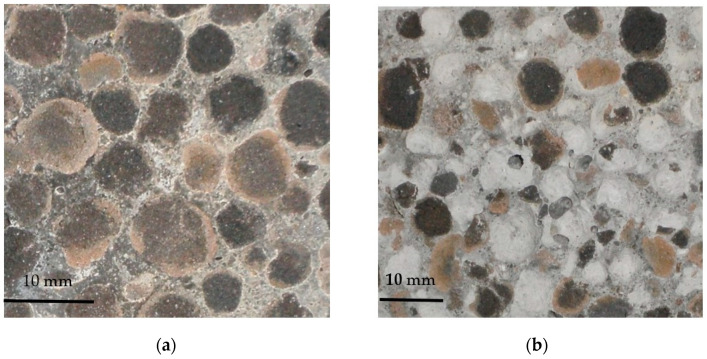
Appearance of a fracture in lightweight concretes with an aggregate (**a**) initially dried—1D and (**b**) initially saturated—1S, subject to splitting.

The mean results for the depth of water penetration under pressure tests are given in Figure 4. The appearance of split specimens after water permeability tests is presented in Figure 5 and Figure 6.

The mean values ranged from 10 up to 74 mm. Individual results usually differed from the average values of the water front depth by no more than 10 mm, excluding concretes prepared with pre-saturated aggregates. In these latter cases, the spread of results even exceeded 20 mm. The relatively high resulting dispersion was mostly caused by the specifics of the standard procedure itself. 

The results achieved show that not all tested lightweight concretes may be regarded as resistant to water under pressure. In many European countries, it is assumed that the depth of water penetration in concrete should not exceed 50 or 30 mm when it is exposed to moderate or severe aggression, respectively, e.g., [44,45].

### 3.3. Freeze-Thaw Resistance

**Figure 7:**
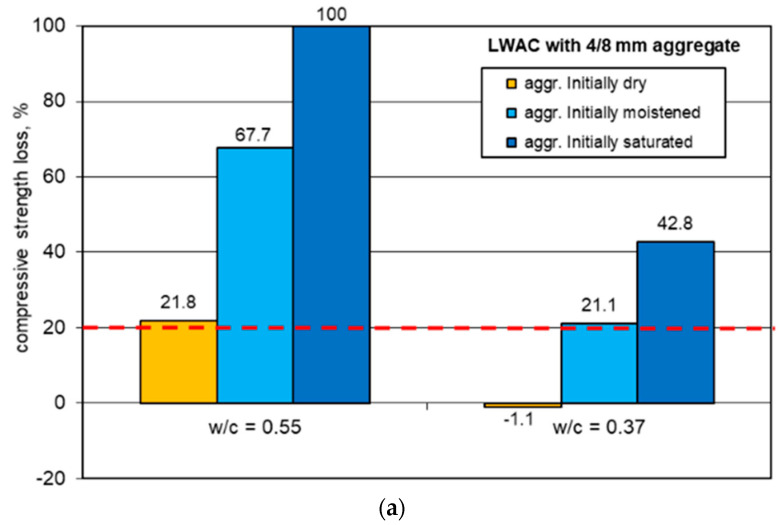

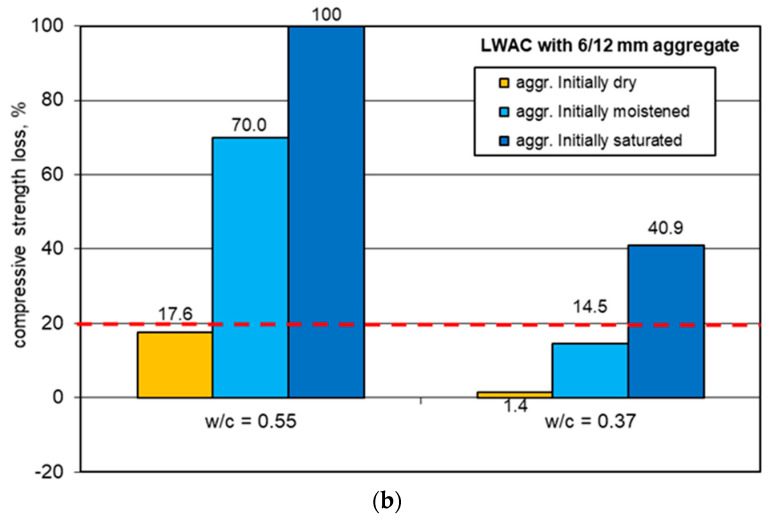
The loss of compressive strength of LWAC after 150 freeze-thaw cycles: (**a**) LWAC with aggregate 4/8 mm and (**b**) LWAC with aggregate 6/12 mm.

**Figure 8:**
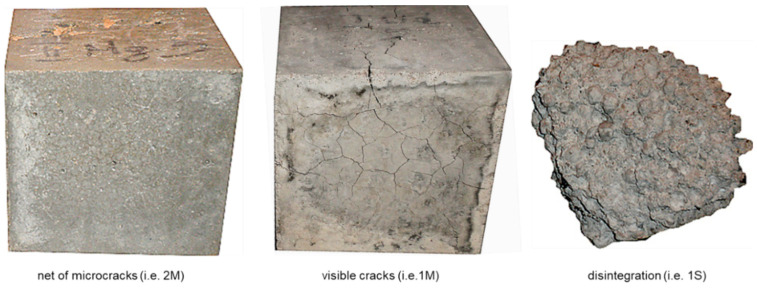
The appearance of LWAC specimens after freeze-thaw cycles.

The results of the freeze-thaw resistance tests are given in Figure 7 and Table 7. The characteristic appearance of specimen damage after freezing and thawing cycles is presented in Figure 8.

The coefficient of variation of compressive strength specified on reference specimens was the same, on average (0.05), as for standard specimens (150 × 150 × 150 mm) referred to in Table 6. Nevertheless, due to the smaller size cubes (100 × 100 × 100 mm) used for the freeze-thaw tests, the dispersion of results for particular concrete series was slightly higher. As a result, the coefficient of variation ranged from 0.03 up to 0.12, irrespective of the initial aggregate moisture. However, in the case of specimens subject to freeze-thaw cycles, the scatter of compressive strength results was significant. The coefficient of variation for concrete series that did not disintegrate before 150 cycles reached up to 0.23 and seemed to be connected to the strength loss. The concrete series revealing higher strength loss due to freeze-thaw cycles also showed a higher dispersion of results. Both of these phenomena can be explained by the more numerous microcracks occurring in these particular series.

The values of compressive strength loss and weight loss ranged from 0% up to 100%. On the one hand, specimens of concretes made of a weaker cement matrix and pre-saturated aggregate (1s and 1S) did not sustain more than 10–30 cycles. On the other hand, concretes made of a stronger cement matrix and initially dry aggregate (2d and 2D) revealed no destruction signs after 150 or 200 cycles of freezing and thawing. In the case of concrete 2d, even negative strength loss (see Table 7) was observed. This indicated the slightly higher mean strength of specimens subject to 150 freeze and thawing cycles in relation to the mean value achieved for reference specimens. Such an observation probably resulted from the simple dispersion of strength results and actually proved no strength loss. In the case of reference specimens of concrete 2d, a much higher coefficient of variation was achieved (0.12) than for the other reference series (0.03–0.07). 

## 4. Discussion

**Figure 9:**
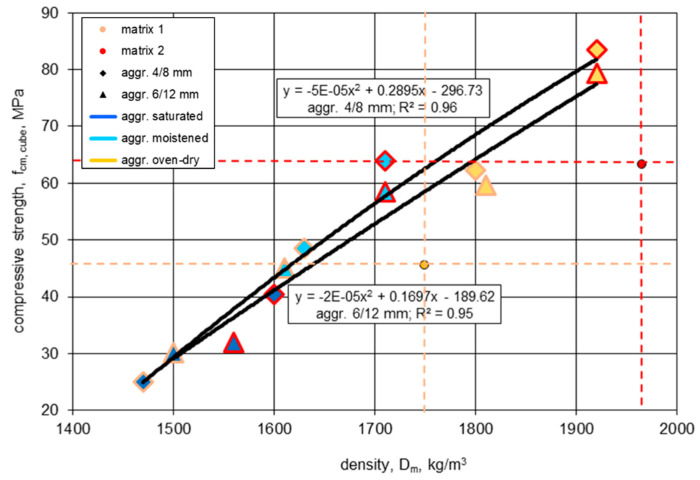
The relationship between mean values of the oven-dried density and compressive strength for lightweight concretes and their cement matrices.

As can be seen in Figure 9, all parameters taken into account, i.e., the nominal w/c, aggregate initial condition, and aggregate type, which affect the actual cement matrix strength, the aggregate strength, and their volume share, were found to influence the strength and density of LWAC. Moreover, a clear relationship between the oven-dried density of tested lightweight concretes and their compressive strength was shown. Generally, the higher the concrete density, the higher the strength. However, a certain influence of the sintered fly ash aggregate size on this relationship was found. The application of the 4/8 mm fraction enabled concrete to achieve a greater strength (6% greater, on average) in relation to the 6/12 mm fraction, while the aggregate size did not affect the density at all. Such a tendency is caused by the slightly higher crushing resistance of the 4/8 mm aggregate at a very similar particle density in comparison with the 6/12 mm fraction.

It should be noted that, despite the application of a very porous aggregate, half of the lightweight concretes, especially those made of an initially dry aggregate, obtained compressive strengths higher than the strength of their cement matrix. This proves the advantageous mechanism of the aggregate absorption of water from cement paste, resulting in a decrease in the water–cement ratio in relation to its nominal value.

As expected, most concretes tested under an oven-dried condition revealed higher compressive strengths than those tested under standard saturated conditions (Table 6). Nevertheless, the difference between the two results, specified under various conditions, ranged from 0% to 18% and seemed to be dependent on the initial moisture content of the aggregate. Therefore, the influence of the moisture content of concretes prepared with a pre-saturated aggregate was not observed at all, while in the case of concretes made of an initially dry aggregate, the influence was the most pronounced. Such an observation may be explained by the different microstructure of concretes prepared with aggregates under various initial conditions (see Section 4.4).

### 4.1. Water Absorption

**Figure 10:**
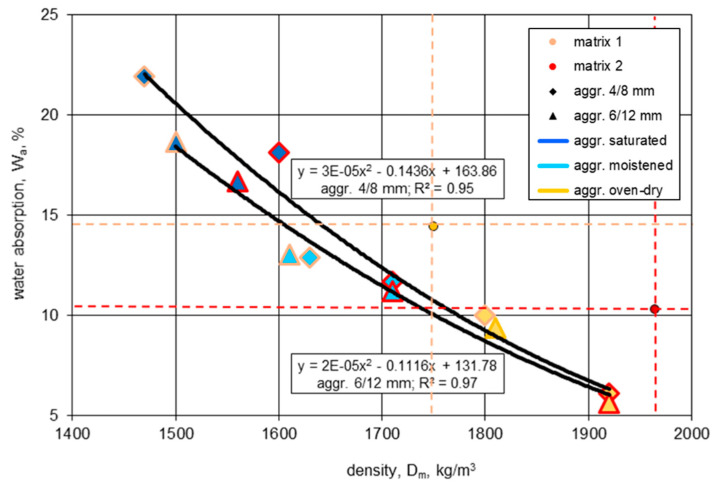
The relationship between the oven-dried density and water absorption for lightweight concretes and their cement matrices.

The water absorption of tested concretes turned out to be strongly dependent on their densities, since it was determined by the porosity of both the aggregate and the cement matrix. The higher the density, the lower the water absorption of the composites (Figure 10). However, the relationship differed slightly for concretes made of aggregates of different sizes. Surprisingly, less water absorption was revealed by concretes prepared with the more porous and weaker 6/12 mm aggregate. This probably resulted from the fact that the 6/12 mm aggregate contained fewer crushed particles than the 4/8 mm aggregate (Figure 1). Such a hypothesis may be proved by the lower absorption of the bigger fraction after saturation in water for more than 72 h (Figure 2).

The results of water absorption, like the results of the strength tests, proved the observation made in [9] for the water absorption mechanism of the lightweight aggregates. Owing to the mechanism, it is possible to make the LWAC structure tighter and stronger. As a result, half of the lightweight concretes, especially those made of an initially dry aggregate, showed even lower water absorption than the matrix used for concrete preparation. 

In spite of the application of the aggregate characterized by high water absorption, it was possible to produce concretes 2d and 2D with water absorption comparable to that typical for normal-weight concretes. The other lightweight concretes revealed water absorption values several times higher than those of normal-weight composites of the same strength. In the case of NWAC, such high water absorption would lead to a very poor durability.

### 4.2. Water Permeability

The results for the depth of water penetration under pressure indicate that lightweight concretes made of both initially dry and moistened aggregates had a sufficient water tightness, even for structures or elements subject to aggressive exposures. In the case of concretes prepared with an initially saturated aggregate, the depth was several times higher than that of the other concretes. Interestingly, depending on whether the aggregate was initially pre-saturated or not, the type of cement matrix and the initial moisture content were less important. The biggest impact of the initial saturation of the aggregate may be explained by the poor adhesion of cement paste to the pre-saturated aggregate. Such a mechanism was proven by the fracture of specimens split after permeability tests (Figure 5 and Figure 6). In the case of concretes prepared with a pre-saturated aggregate, the common path of cracking occurred through the bond between the aggregate and cement paste. Meanwhile, for concretes made from an initially dry aggregate, this mode of fracture did not occur, and for concretes prepared with an initially moistened but not fully saturated aggregate, it was very rare. This may be explained not only by the lower share of mortar in concretes made with saturated aggregates (a higher matrix content would lead to the segregation of components), but above all, by the higher porosity and more numerous micro-cracks in the transition zone. Regardless of the w/c, the tested concretes with pre-saturated aggregate showed almost identical penetration depths. This suggests that the penetration of water under pressure occurs in the least tight areas which, in this case, was mainly the contact zone and not the matrix itself.

In contrast to water absorption tests, there was no direct relationship between the depth of water penetration in lightweight concretes and their density or strength. Therefore, there was no relationship between the water permeability tested according to EN 12390-8 [40] and the water absorption. A relatively high water absorption of the tested lightweight concretes, even up to 13%, cannot be identified with poor water tightness.

The results of this research did not confirm the statement contained in [21], that there is an optimal range of cement content (ca 500 to 600 kg/m^3^) that promotes water tightness in LWAC. The achieved results showed that lightweight concretes containing even a moderate cement content, i.e., 336 kg/m^3^, may have a relatively low depth of water penetration, comparable to concretes made of a much higher cement content than 500 kg/m^3^. Moreover, in contrast to the studies described in [17,19,20], this research revealed that even when aggregates with relatively greater water absorption (WA_24h_ > 10–15%) and cement matrixes with a relatively higher water–binder ratio (>0.4) are applied, it is possible to produce lightweight concretes with a comparatively low water permeability. All tested lightweight concretes, except for those made with a pre-saturated aggregate, exhibited little water permeability, comparable or slightly lower than typical for normal-weight concretes of a similar strength. 

### 4.3. Freeze-Thaw Resistance

The freeze–thaw test results obtained proved statements presented in [10,18,27,28,29] indicating that lightweight concretes, even when air entraining admixtures are not used, may be characterized by a high frost resistance. Nevertheless, the possibility of the production of LWAC with a higher or comparable freeze-thaw resistance to NWAC is dependent on both material and technological factors.

The conducted tests clearly show that lightweight concretes with a pre-saturated sintered fly ash aggregate, regardless of its size and nominal water–cement ratio, did not meet the freeze-thaw resistance criteria specified in PN-B-06265 [42]. The appearance of specimens 1s and 1S after the freezing and thawing cycles (Figure 8) indicated that the early structural disintegration of concretes made of a pre-saturated aggregate is mostly caused by poor bonding between aggregate particles and the cement matrix. In the case of concretes made of an initially saturated aggregate and stronger matrix (2s and 2S), which, after 150 cycles, kept their integrity but revealed visible cracks, the bonds between the aggregate and cement matrix represented the weakest zone of the concrete structure. During the compressive strength test, the specimens mostly cracked through the contact zone, which is not typical behavior of LWAC.

The reduction of the aggregate initial moisture content and the water–cement ratio caused an increase in the freeze-thaw resistance of tested concretes. As a result, when using a pre-dry aggregate and tighter matrix (w/c = 0.37), no signs of damage or decrease in strength were observed, not only after 150 cycles, but also after 200 cycles. The results proved the observation made in [27,32], that the application of an initially dry aggregate may lead to a comparable or much better frost resistance of lightweight concretes compared to ordinary concretes of a similar volume composition. 

There is also a certain impact of the applied aggregate fraction on the freeze-thaw resistance. The application of the weaker 6/12 mm fraction containing fewer crushed particles turned out to be more advantageous in terms of the LWAC freeze-thaw resistance. Therefore, initially stronger concretes made of the 4/8 mm fraction revealed slightly higher strength loss after freeze-thaw cycles in comparison to LWAC with the 6/12 mm fraction.

It should be emphasized that no direct relationship between the strength and freeze-thaw durability was observed. For example, reference specimens of concretes 1M, 2s, and 2M showed very similar compressive strength values: 49.4, 49.5, and 52.5 MPa, respectively. However, they revealed completely different strength losses after freezing and thawing cycles, i.e., 70.0%, 42.8%, and 14.5%, respectively. Similarly, there was no direct relationship, as indicated in [3], between the cement content in LWAC and its freeze-thaw resistance. For example, concrete 2M containing 386 kg/m^3^ of cement showed less strength loss after 150 cycles (14.5%) than concrete 1D containing 508 kg/m^3^ of cement (17.6%).

The condition for obtaining a higher freeze-thaw resistance of LWAC in relation to NWAC (a degree of aggregate pre-wetting lower than 90% of the total porosity of the concrete), presented in [28], turned out to be insufficient for the tested lightweight concretes. It should be stated that the application of the sintered fly ash aggregate initially moistened to ca. 70% of its water absorption, which is less than 90% of the total concrete porosity, only provided a freeze-thaw resistance of LWAC comparable to NWAC with a similar compressive strength in the case when the aggregate did not contain too many crushed particles and a tighter cement matrix (w/c = 0.37) was used. However, the application of an initially dry aggregate, depending on the type of cement matrix, ensured a similar or much better freeze-thaw resistance than for normal-weight concretes, despite the much higher water absorption of LWAC, even when air-entraining admixtures were not used.

### 4.4. Microstructure Analysis

In order to verify the hypothesis of the key importance of the lightweight aggregate’s initial moisture condition for the durability of the tested LWAC, an SEM analysis was carried out for selected concretes.

**Figure 11:**
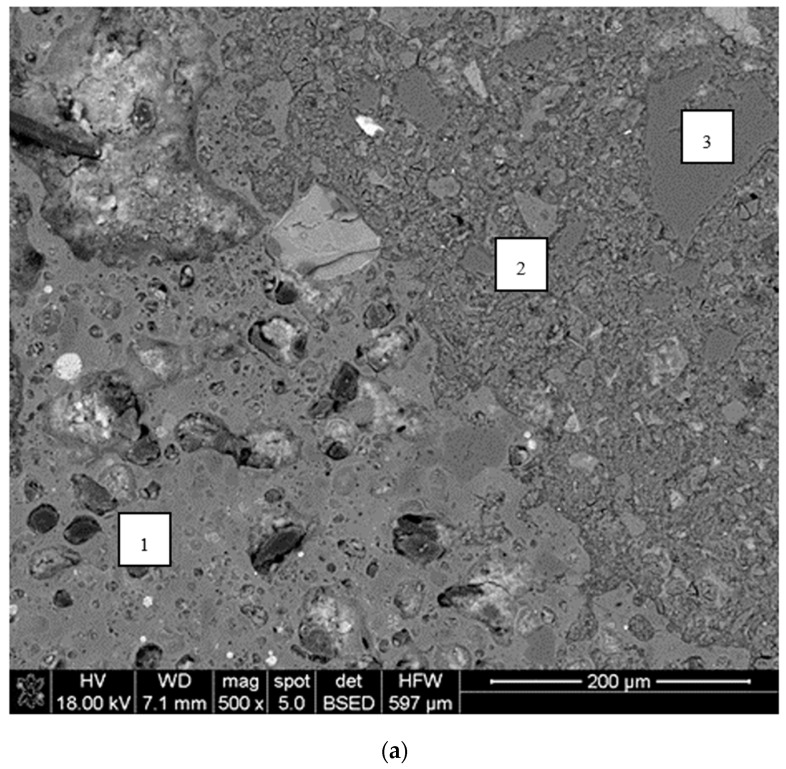

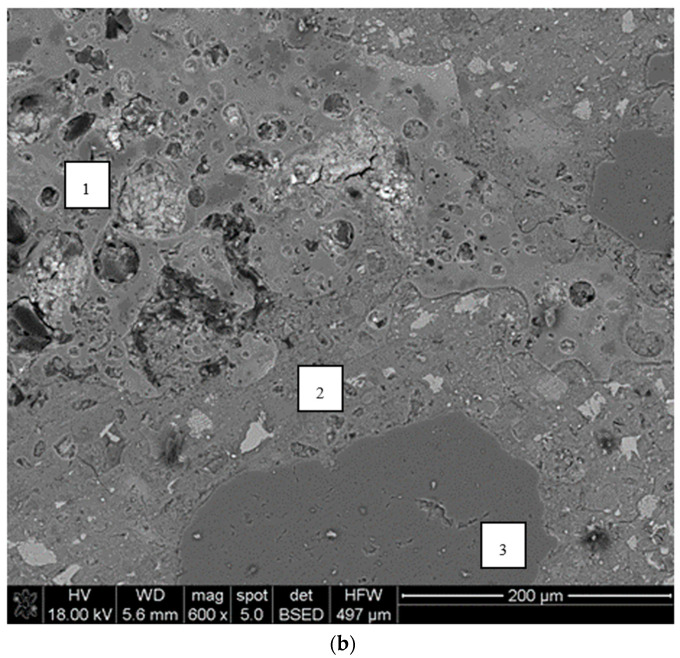
Interfacial transition zone in lightweight concretes with an aggregate (**a**) initially saturated—1S and (**b**) initially dried—1D (SEM: ×600); 1—LWA, 2—cement paste, and 3—sand particle.

Generally, the adhesion between cement paste and sintered fly ash particles may be assessed as very good, irrespective of the initial moisture conditions of the lightweight aggregate (Figure 11). As can be seen in Figure 11a, the cement paste tightly filled all external pores of the sintered fly ash particle, even when the aggregate was initially saturated. Such an observation is consistent with the statement presented in [4], that the initial moistening of LWA has no impact on the depth of cement paste migration into the aggregate particles. The cement paste was observed, even in very deep pores, to be apparently isolated from the LWA shell. This indicates that the interlocking mechanism of the LWAC bond can be explained by the mixing of water accommodated in porous aggregates with cement paste, rather than by the absorption of cement paste itself by the aggregate.

There were some basic differences in the appearance of specimens of concretes made of sintered fly ash aggregates under various initial moisture conditions. Firstly, concretes made of aggregates with an initially lower moisture content revealed a higher content of unhydrated cement particles. The difference was especially visible when comparing concretes prepared with pre-dried and pre-saturated aggregates (Figure 11). The lower degree of hydration observed in the case of concretes with aggregates with a lower initial moisture content proved the effectiveness of the absorption mechanism of the porous aggregate in the reduction of the cement matrix water–cement ratio. Secondly, the structure of cement paste in concretes prepared with aggregates with a lower initial moisture content was tighter due to their lower porosity and considerably smaller number of microcracks.

**Figure 12:**
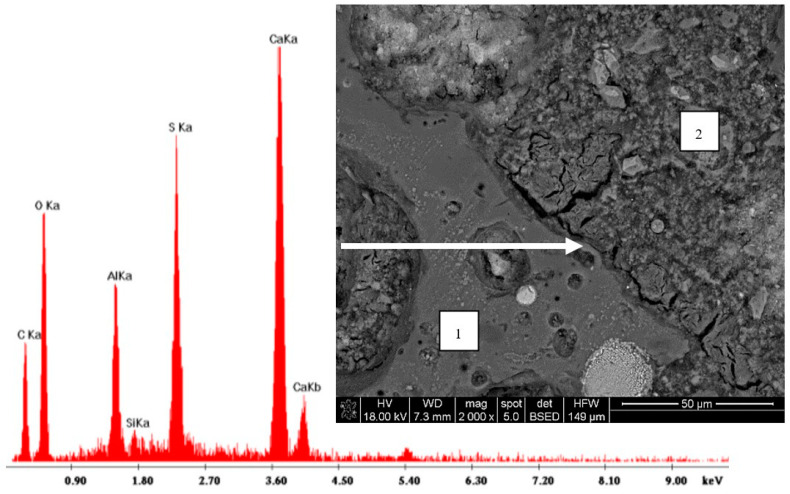
Microanalysis and image of ettringite in the interfacial transition zone (ITZ) of lightweight concrete with initially saturated aggregate (1S); 1—LWA and 2—cement paste.

The analysis of the cement paste structure under larger magnification, as well as the EDS analysis of concretes with pre-saturated aggregates, showed that the visible microcracks were mainly connected to ettringite formation (Figure 12). In particular, many areas of an increased ettringite content and assisting microcracks were observed in the interfacial transition zone (ITZ). This observation is in contradiction to the models of the interfacial transition zone presented in [10], where no ITZ was assumed for saturated sintered fly ash aggregate concrete. Meanwhile, in this research, the concretes prepared with aggregates with a lower initial moisture content usually did not reveal a specific ITZ characterized by a higher porosity and a higher number of ettringite formations at all. As a result, concretes with pre-saturated aggregates, when they were subject to strength tests or freeze-thaw cycles, showed a visibly lower durability and a fracture path thorough the bonds between aggregates and cement paste. 

The analysis carried out showed that the general conclusion presented in the review [10], that the thickness and the quality of the ITZ of a sintered fly ash aggregate are superior to normal aggregate concrete, did not turn out to be valid in the case of the tested LWAC with an initially saturated aggregate. However, the microstructure of the interfacial transition zone may be a reliable indicator of the durability of LWAC. A tight and homogenous ITZ was observed in durable lightweight aggregate concretes. 

## 5. Conclusions

The tests carried out and the analysis of the achieved results showed that the process of the formation of LWAC’s durability is much more complex than in the case of NWAC. While the durability of normal-weight concrete is mainly determined by the tightness of the cement matrix and its adhesion to aggregates, for lightweight concretes, in addition to these two factors, the properties of the applied aggregate and technological procedures have extremely important significance. In particular, the following conclusions can be made:

- The lightweight aggregate water absorption and its initial moisture content influence the concrete durability to a large extent. Nevertheless, even the application of LWA with water absorption as high as ca 25% makes it possible to produce durable concrete. However, in the case of such an aggregate, the procedure of LWA initial pre-saturation should not be allowed in practice;
- Regardless of the nominal water–cement ratio or cement content, concretes made of the initially saturated sintered fly aggregate revealed very high levels of water absorption (up to 22%), an unacceptable depth of water penetration under pressure (up to 74 mm), and a lack of freeze-thaw resistance;
- Limiting the initial sintered fly ash moisture content to 17–18% enhanced the concrete water tightness considerably, but it was not able to ensure a good freeze-thaw resistance. To make such concrete resistant to freezing–thawing cycles, it is also necessary to limit w/c and apply an LWA fraction not containing too many crushed particles;
- The application of the initially dry sintered fly ash aggregate and cement matrix with a relatively low nominal water–cement ratio (w/c = 0.37) led to a comparatively low LWAC water absorption and permeability, as well as a complete freeze-thaw resistance, even without air entraining. The matrix’s volume share, cement content, and even concrete strength were found to be of secondary importance;
- The microstructure of the interfacial transition zone may be a reliable indicator of the durability of LWAC. A tight and homogenous ITZ was observed in durable lightweight aggregate concretes, especially those made of an initially dry aggregate. In the case of the application of a pre-saturated aggregate, the interfacial transition zone was characterized by a high content of ettringite and accompanying microcracks resulted in a poor concrete durability;
- There are no direct relationships among factors commonly considered to affect concrete’s durability, i.e., the water absorption, compressive strength, or cement content and permeability, and freeze-thaw resistance of the tested LWAC with a sintered fly ash aggregate. Generally, the much greater water absorption of LWAC should not be identified with its lower durability in comparison to NWAC.

## Figures and Tables

**Table 1:** Properties of sintered fly ash aggregates used for lightweight aggregate concrete (LWAC).

Fraction	Specific Density, kg/m^3^	Particle Density, kg/m^3^	Bulk Density, kg/m^3^	Water Absorption after 24 h, %	Max. Water Absorption, %	Crushing Resistance, MPa
4/8 mm	2490	1320	730	19.3	25.3	8.0
6/12 mm	2490	1340	720	18.8	24.3	7.2

**Table 2:** Chemical composition of the cement and lightweight aggregate used for LWAC.

Component	CaO, %	SiO_2_, %	Al_2_O_3_, %	Fe_2_O_3_, %	SO_3_, %	MgO, %	Na_2_O_eqv_., %	Loss of Ignition, %
CEM I 42,5R	63.6	22.1	5.6	3.1	2.6	1.2	0.8	0.9
Lytag	2.2	58.0	22.0	3.1	0.3	1.4	0.9	<4

**Table 3:** Parameters of sintered fly ash aggregate concretes.

Mix Designation	Nominal w/c	LWA Fraction, mm	LWA Initial Moisture Content, %	LWA Volume Share, %
1d	0.55	4/8	0.0	42
1m	0.55	4/8	17.0	59
1s	0.55	4/8	25.3	68
1D	0.55	6/12	0.0	46
1M	0.55	6/12	17.7	61
1S	0.55	6/12	24.3	67
2d	0.37	4/8	0.0	41
2m	0.37	4/8	17.0	58
2s	0.37	4/8	25.3	70
2D	0.37	6/12	0.0	43
2M	0.37	6/12	17.7	60
2S	0.37	6/12	24.3	68

**Table 4:** Compositions of sintered fly ash aggregate concretes.

Mix Designation	LWA ^1^, kg/m^3^	Natural Sand, kg/m^3^	Cement, kg/m^3^	Water, kg/m^3^	Superplasticizer, kg/m^3^
1d	572	619	516	284	0.0
1m	950	406	338	186	0.0
1s	1171	288	239	132	0.0
1D	603	610	508	279	0.0
1M	945	404	336	185	0.0
1S	1110	306	225	140	0.0
2d	559	700	584	216	14.6
2m	935	463	386	143	9.6
2s	1191	322	268	99	7.0
2D	569	666	555	205	13.9
2M	925	462	386	142	9.6
2S	1129	346	288	107	7.2

^1^ Lightweight coarse aggregate under initial moisture conditions.

**Table 5:** Test type, time, and procedure, and specimen type and number of tests.

Test	Specimens	Specimens Number	Concrete Age	Procedure
Density	Cube 150 mm	3	28 days	EN 12390-7 [38]
Compressive Strength	Cube 150 mm	6	28 days	EN 12390-3 [39]
Water Permeability	Cube 150 mm	6	28 + 7 days	EN 12390-8 [40]
Water Absorption	Cube 150 mm	3	28 days	PN-88/B-06250 [41]
Freeze-Thaw Resistance	Cube 100 mm	12	28 + 7 days	PN-B-06265 [42]

**Table 6:** Mean values of the basic properties of sintered fly ash aggregate concretes, determined at 28 days.

Mix Designation	D_m_^1^, kg/m^3^	D_m_^2^, kg/m^3^	f_cm_^1^, MPa	S_f_^1^, MPa	f_cm_^2^, MPa	S_f_^2^ MPa	WA_m_, %	S_WA_, %
1d	2160	1800	56.1	2.4	62.3	3.8	10.0	0.05
1m	2000	1630	45.6	2.5	48.5	2.2	12.9	0.05
1s	1810	1470	25.1	1.8	25.0	1.7	21.9	0.05
1D	1990	1820	53.2	2.9	59.6	3.0	9.4	0.00
1M	1990	1620	42.1	2.3	45.0	2.0	13.0	0.09
1S	1930	1500	30.3	1.9	30.1	2.2	18.7	0.05
2d	2040	1920	71.0	3.3	83.5	3.8	6.1	0.08
2m	2110	1720	59.5	2.5	64.0	3.6	11.7	0.09
2s	2050	1600	40.8	2.8	40.4	3.0	18.1	0.05
2D	2030	1920	69.8	3.2	79.4	2.9	5.6	0.05
2M	2100	1720	53.4	2.5	58.5	2.9	11.2	0.09
2S	1980	1560	32.3	2.2	32.0	2.0	16.7	0.12
matrix 1	2040	1750	43.2	2.1	45.5	2.3	14.4	0.05
matrix 2	2160	1970	60.1	3.3	63.3	3.3	10.3	0.09

D_m_—mean density; f_cm_—mean compressive strength; S_f_—standard deviation for compressive strength results; WA_m_—mean water absorption; S_WA_—standard deviation for water absorption results; ^1^ determined under saturated conditions; ^2^ determined under oven-dried conditions.

**Table 7:** The results of freeze-thaw tests of sintered fly ash aggregate concretes.

Mix Design	f_cm1_, MPa	S_f1_, MPa	f_cm2_, MPa	S_f1_, MPa	(f_cm1_ − f_cm2_)/f_cm1_, %	Weight Loss, %	Specimens’ Condition after 150 (200 *) Freezing–Thawing Cycles
1d	65.1	2.2	50.9	5.8	21.8	3.4	net of microcracks or no cracks
1m	56.7	1.8	18.3	2.0	67.7	7.5	visible cracks
1s	31.5	2.2	0.0	-	100	100	disintegration after 10–30 cycles
1D	58.5	4.0	48.2	4.5	17.6	2.5	net of microcracks or no cracks
1M	49.4	1.6	14.8	3.4	70.0	5.3	visible cracks
1S	34.3	2.4	0.0	-	100	100	disintegration after 10–30 cycles
2d	72.3	8.6	73.1	4.2	−1.1	−0.5	no cracks
2d *	74.4	2.7	73.9	3.5	0.7	0.2	no cracks
2m	65.5	3.5	51.7	7.0	21.1	4.7	net of microcracks or no cracks
2s	49.5	1.3	28.3	6.6	42.8	7.4	net of microcracks or visible cracks
2D	71.3	3.3	70.3	7.5	1.4	−0.5	no cracks
2M	52.5	3.9	44.9	6.1	14.5	1.4	net of microcracks or no cracks
2S	36.2	1.3	21.4	4.7	40.9	2.6	net of microcracks or visible cracks

f_cm1_—mean compressive strength determined on reference specimens under saturated conditions not subject to freezing and thawing cycles; S_f1_—standard deviation for f_cm1_; f_cm2_—mean compressive strength determined on specimens under saturated conditions subject to freezing and thawing cycles; S_f2_—standard deviation for f_cm2_; * 200 freezing–thawing cycles were carried out only for concrete 2d (explanation in the text).
